# The OASI care bundle quality improvement project: lessons learned and future direction

**DOI:** 10.1007/s00192-021-04786-y

**Published:** 2021-05-14

**Authors:** Magdalena Jurczuk, Posy Bidwell, Ipek Gurol-Urganci, Jan van der Meulen, Nick Sevdalis, Louise Silverton, Ranee Thakar

**Affiliations:** 1 Centre for Quality Improvement and Clinical Audit, Royal College of Obstetricians and Gynaecologists, 10–18 Union Street, London, SE1 1SZ UK; 2 Department of Health Services Research and Policy, London School of Hygiene and Tropical Medicine, 15–17 Tavistock Place, London, WC1H 9SH UK; 3 Centre for Implementation Science, Health Service and Population Research Department, King’s College London, De Crespigny Park, London, SE5 8AF UK; 4 Royal College of Midwives, 10–18 Union Street, London, SE1 1SZ UK; 5 Croydon University Hospitals NHS Trust, 530 London Road, Croydon, CR7 7YE UK

**Keywords:** OASI Care Bundle, Obstetric anal sphincter injury, Severe perineal tear, Scale-up, Quality improvement, Implementation

## Abstract

Rising rates of obstetric anal sphincter injury (OASI) led to a collaborative effort by the Royal College of Obstetricians and Gynaecologists (RCOG) and the Royal College of Midwives (RCM) to develop and evaluate the OASI Care Bundle (OASI-CB). The OASI-CB comprises four practices (antenatal discussion about OASI, manual perineal protection, mediolateral episiotomy at 60° from the midline, and systematic examination of the perineum, vagina and ano-rectum after vaginal birth) and was initially implemented as part of a quality improvement (QI) project—“OASI1”—in 16 maternity units across Great Britain. Evaluation of the OASI1 project found that the care bundle reduced OASI rates and identified several barriers and enablers to implementation. This paper summarises the key findings, including strengths, limitations and lessons learned from the OASI1 QI project, and provides rationale for further evaluation of the OASI-CB.

## Introduction

The threefold rise in the reported rate of obstetric anal sphincter injury (OASI) in England between 2000 and 2011 [1], with one in 16 primiparous women sustaining an OASI [2], called into question whether sufficient action was being taken to prevent this severe complication of vaginal birth. OASI can have long lasting consequences on women’s continence, sexual function, and mental health, all of which significantly impact on quality of life [3]. More than half of women with OASI experience ongoing symptoms and approximately half report an impact on their future birth choices [3, 4].

Obstetric anal sphincter injuries have significant resource implications for healthcare providers owing to the ongoing follow-up [5] and can trigger negligence claims [6]. The total value of OASI-related negligence claims in the National Health Service (NHS) in England was an estimated £31.2 million between 2000 and 2010. These claims referred specifically to failure to perform or extend an episiotomy, failure to diagnose the true extent and grade of the injury, inadequacy of repair and failure to perform a repair [6].

Globally, there have been significant efforts to prevent OASI. Evidence from several countries shows reductions in OASI rates through targeted quality improvement (QI) initiatives [7–10]. One such initiative is the “OASI Care Bundle” (OASI-CB), a collaborative effort to standardise maternity care related to OASI prevention led by the Royal College of Obstetricians and Gynaecologists (RCOG) and the Royal College of Midwives (RCM).

In this paper, we summarise the development of the OASI-CB, and discuss the clinical and implementation outcomes from the OASI-CB QI Project, referred to hereafter as “OASI1”. We also reflect on the lessons learned and provide rationale for further evaluation of the OASI-CB.

## Development of the OASI-CB 2014–2016

In 2014, the RCOG and the RCM convened a working group comprising expert obstetricians, midwives, and methodologists to discuss how to address the impact of OASI. The group reviewed national and international initiatives to reduce perineal trauma and agreed that a “care bundle” of interventions would be the appropriate course of action. A care bundle is “a small set of evidence-based practices that, when implemented together result in significantly better outcomes than when implemented individually” [11].

**Fig. 1:**
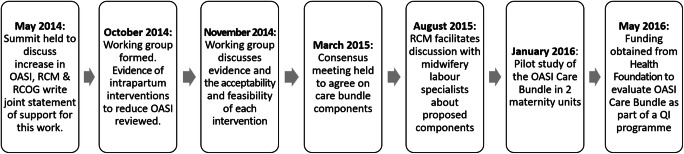
Development of the Obstetric Anal Sphincter Injury Care Bundle (OASI-CB) and the beginning of the OASI1. *RCM* Royal College of Midwives, *RCOG* Royal College of Obstetricians and Gynaecologists, *QI* quality improvement

In March 2015, the working group and a lay member of the RCOG’s Women’s Network convened to review evidence from national guidelines, randomised controlled trials (RCTs) and key observational studies of intrapartum care interventions to reduce OASI. The interventions that were discussed covered six themes: episiotomy, perineal protection, maternal position, pushing technique and coaching, instrument choice and “other” (which included pain relief, water birth, hyaluronidase and use of devices such as EpiScissors). Overview of the evidence highlighted that most RCTs that reported OASI outcomes lacked sufficient statistical power to evaluate the intervention’s impact on OASI rates, and meta-analyses of RCTs within each theme were difficult to interpret owing to significant heterogeneity in the definition of the interventions included. The OASI-CB elements were selected following consideration of various definitions of interventions, the quality of evidence, as well as the feasibility for their inclusion into a standardised care bundle. The OASI-CB was further refined following an RCM consultation with labour ward specialists, managers and educationalists, which resulted in an emphasis on clinicians’ engagement with women, mobility during labour and facilitating chosen birth positions (Fig. 1).

**Fig. 2:**
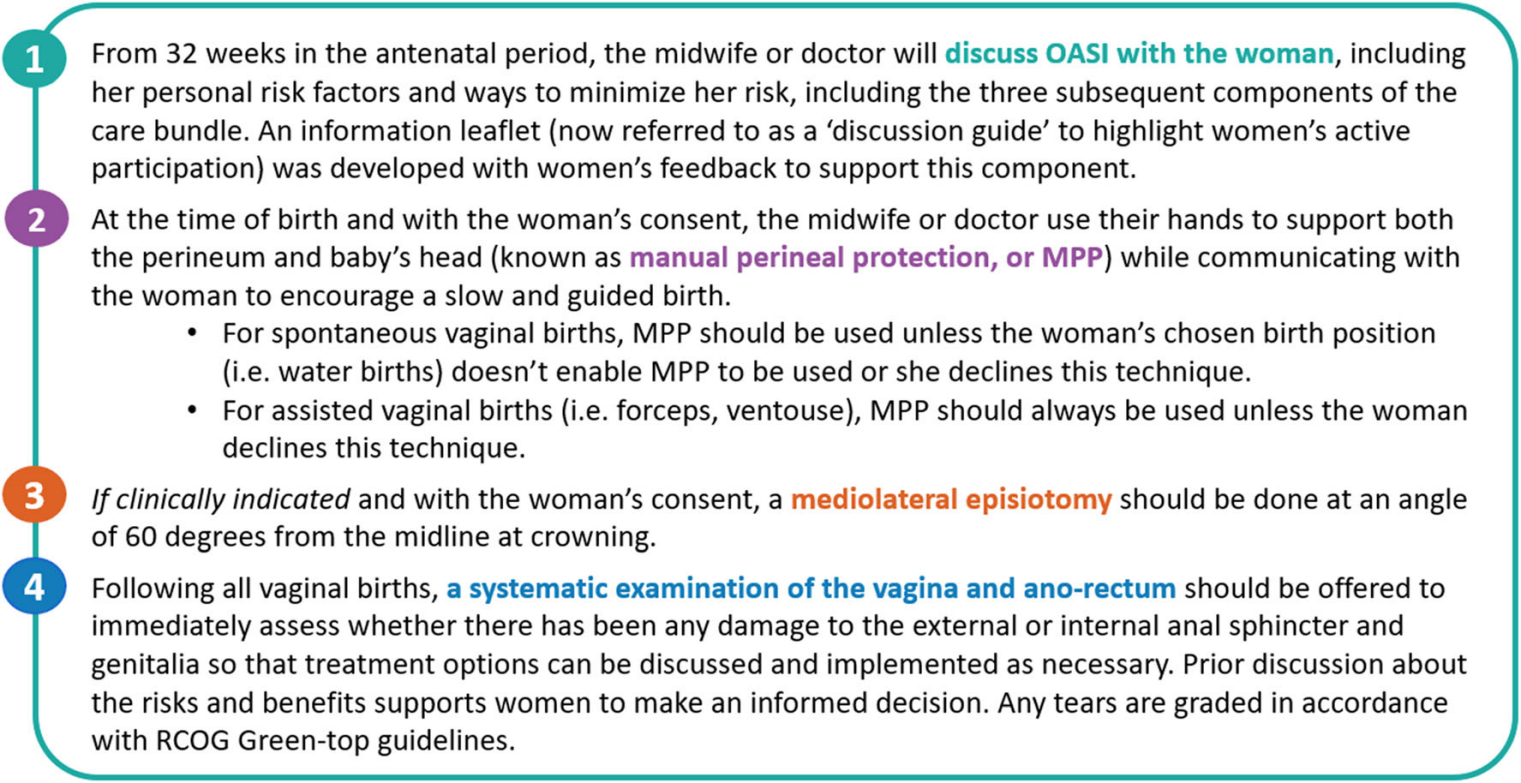
The final four components of the Obstetric Anal Sphincter Injury Care Bundle (OASI-CB). *RCOG* Royal College of Obstetricians and Gynaecologists

The final four components of the OASI-CB are in Fig. 2, all underpinned by good communication with the woman and her informed consent.

The high quality of evidence supporting the efficacy of warm compresses [12] triggered much discussion about their inclusion in the bundle. However, it was decided that the variation in utilisation and clinical practicalities (whether the compress is held continuously, what materials are used, the temperature, and the feasibility of safely heating/reheating compresses) made it unfeasible to include as a standardised component of the bundle. Use of warm compresses was encouraged as part of intrapartum care in units with local protocols to support this.

## Overview of OASI1

In 2016, the OASI-CB was successfully piloted in two NHS maternity units over a 3-month period, demonstrating the care bundle’s acceptability to clinicians and feasibility of implementation in clinical practice. Funding was subsequently obtained from the Health Foundation to implement OASI1. OASI1 was supported by an Independent Advisory Group (IAG), which included national experts, women’s organisations and lay representatives. The group met biannually to review progress and reflect on the implementation process and initial findings. Members of the IAG and women from stakeholder organisations contributed to discussion panels at the project’s final Dissemination Event (November 2018).

OASI1 evaluated the clinical effectiveness of the OASI-CB using a multicentre stepped wedge cluster design in 16 maternity units in four regions across Great Britain from 2016 to 2018 [13]. Implementation of the care bundle was through a stepwise regional roll-out every 3 months starting in January 2017 and was led locally by midwives and obstetrician champions from each maternity unit. Sustained leadership and support were provided by the RCOG, RCM and the Project Team in the form of facilitated skills training sessions, an awareness campaign and supervisory visits.

The primary clinical outcome evaluated in OASI1 was the rate of OASI prior to and after implementation of the OASI-CB. Secondary clinical outcomes evaluated were episiotomy and caesarean birth rates. The clinical evaluation relied on routinely collected maternity data [14]. Secondary outcomes related to implementation of the care bundle, namely acceptability, coverage, feasibility and sustainability, were studied qualitatively through interviews and focus group discussions with clinicians and women who received the care bundle during birth. A qualitative exploration of clinicians’ and women’s perspectives of the care bundle enabled us to identify enablers and barriers to implementation [15, 16].

## Reflecting on the results, strengths and limitations of OASI1

### Overview of results and strengths

#### Demonstrated effectiveness in reducing OASI rates

OASI1 demonstrated the care bundle’s potential for reducing perineal trauma during childbirth. The clinical outcomes evaluation including 55,060 singleton, live, vaginal births found a reduction of 20% in the risk of OASI after the introduction of the care bundle (adjusted odds ratio 0.80, 95% confidence interval 0.65–0.98) [14]. The adjusted odds ratio represents relative differences in the odds of OASI before and after implementation of the care bundle, after accounting for time trends and case-mix factors (age, ethnicity, body mass index, parity, birthweight and mode of birth). The implementation of the care bundle did not affect caesarean section or episiotomy rates.

#### Qualitative exploration of barriers and enablers to implementation

Focus group discussions were held with local clinical champions at the end of the implementation phase to understand barriers and enablers to implementation. Key enablers to implementation included observing positive outcomes related to care bundle use, organisational support, and an increased cohesion between midwives and obstetricians. The main barriers that surfaced were a lack of perineal management skills, resistance to change/ standardisation, and a reluctance to discuss perineal trauma with women in the antenatal period, as this was perceived to cause anxiety to women.

#### Adoption of the OASI-CB by clinicians

The qualitative process evaluation focused on the acceptability, feasibility, coverage and sustainability of the OASI-CB [17]. Sixteen focus groups involving a total of 101 participants explored clinicians’ attitudes towards the care bundle to understand the factors that affected its adoption into their clinical practice. We found that adoption of the OASI-CB was influenced by four main factors, summarised in terms of corresponding barriers and enablers in Table 1.

**Table 1:** Factors that impact clinicians’ adoption of the obstetric anal sphincter injury (OASI-CB)

Factor impacting adoption	Related barrier	Related enabler
How the OASI-CB was introduced/implemented in units	Not all clinicians were consulted about the OASI-CB prior to its roll-out	A well-advertised launch
		Dedicated time for training
		Presence of in-house clinical champions
Opportunities to use the OASI-CB	Presence of student midwives	Involvement of “everyone on the shop floor”
	Clinical practice change takes time	
Receptiveness to change	Conflicting views regarding evidence supporting the OAS-CB	Perceptions of the OASI-CB as a standardised approach to preventing OASI
	Feelings that the OASI-CB challenges professional autonomy	
Perceptions of “what women want”	Associating the OASI-CB with overmedicalisation	Belief that women should be informed and that good communication is key to using the care bundle
	Generalisation of personal reluctance to certain interventions	
	Belief that information about OASI is too scary for women	

#### Acceptability of the OASI-CB to women

Nineteen women were interviewed to learn about their experiences with the OASI-CB. Thematic analysis identified three themes:

1. Memories of touch, whereby women reported that a “hands-on” approach to perineal protection was a positive experience
2. Midwife as a supportive guide, where women reported that good communication facilitated a calm birth and enabled post-birth diagnosis
3. Education: women need more information about perineal trauma

This study indicated that interviewed women did not experience any of the care bundle components as an intrusion of their physical integrity. Additionally, an urgent need was identified for more information about perineal trauma, in terms of risk, prevention and recovery [16].

#### Support of women’s birth choices

The potential misconception that applying MPP during birth restricts women’s mobility and/or their choice of birth position was addressed in OASI1. MPP can be performed in most birthing positions in which women feel comfortable. Although clear visualisation of the perineum at crowning is necessary for MPP to be performed, lack of visualisation is not a reason to restrict a woman’s movement throughout the second stage of labour. The facilitators of the skills training sessions demonstrated how clinicians can adjust their own position to optimise visualisation of the perineum, which was also shown in the manual. The guiding principle for birth position in the second stage of labour is maternal comfort and supporting mobility as well as the widening of the pelvic outlet to assist birth [18].

#### Strong engagement with stakeholder groups

The Project Team worked closely with two stakeholder groups who advocate for women: the Mothers with Anal Sphincter Injuries in Childbirth (MASIC) Foundation and the Birth Trauma Association. The two lay representatives of the OASI1 IAG oversaw the governance and study design. Additionally, the patient and public involvement (PPI) team at the RCOG supported women’s direct involvement throughout the different stages of the project, particularly in the development of the informational leaflet to support the first component of the care bundle.

Implementation of the care bundle was supported by an awareness campaign that included a series of posters displayed in participating maternity units and animated videos featuring experiences of three women with OASIs (available here: https://www.rcog.org.uk/en/guidelines-research-services/audit-quality-improvement/oasi2/videos/). Close collaboration with stakeholder organisations helped to incorporate women’s voices throughout the campaign. Women with OASI also spoke about their experiences at OASI-CB skills training sessions for clinicians facilitated by the Project Team.

#### Expert input and leadership throughout the project

The Project Team’s implementation efforts, such as the delivery of skills training sessions and development of the OASI-CB manual, were informed by clinical expertise from the RCOG and RCM. The OASI1 study design, implementation and evaluation were underpinned by methodological expertise from the London School of Hygiene and Tropical Medicine (LSHTM), and King’s College London in the areas of epidemiology, health services research and implementation science.

### Limitations of OASI1

OASI1 had limitations. We outline these below:

1. Reliance on routinely collected data: clinical outcomes were evaluated using only routinely collected data, which had its limitations. For example, the OASI1 could not measure “coverage” (compliance rate for all eligible births) and “fidelity” (extent to which the care bundle components were applied as intended). Availability of data on OASI-CB coverage and fidelity would have allowed for an analysis of the relative impact of the different clinicians’ compliance with the care bundle on OASI risk reduction. Additionally, varying data completeness and quality of the routinely collected data across participating units precluded assessment of the impact of the OASI-CB on other clinical outcomes of interest (such as anterior tears) or control for some of the main risk factors for OASI (such as shoulder dystocia, epidural use and length of the second stage of labour).
2. Implementation analysis relied on qualitative methods: findings related to the implementation of the care bundle may be limited by the nature of the qualitative methodology used. A recognised limitation of focus groups is that although the dynamic interaction can stimulate thoughts, they can also inhibit participants from divulging their true opinions. Good facilitation can address the potential for socially desirable responses, although it does not eliminate the possibility. Clinicians and women participants in focus groups and interviews respectively were volunteers, which introduces self-selection bias. In addition, interviews with women were conducted approximately 6 weeks post birth; thus, recall bias is possible, although evidence suggests that recall following birth might remain accurate for a long period of time [19]. The small number of interviews offers limited representation of women’s perspectives.
3. Resource-intensive implementation strategies: the enhanced implementation support offered to all OASI1 units by the Project Team and supporting professional organisations could be considered a significant limitation of the OASI1 project in that it does not address the OASI-CB’s scalability. “Scalability” has been defined as “the ability of an intervention shown to be efficacious on a small scale or under controlled conditions to be expanded under real-world conditions to reach a greater proportion of the eligible population, while retaining effectiveness” [20]. The OASI1 project found the OASI-CB to be effective when implemented with intensive support in a relatively small number of units. It is also possible that the nature of the study’s stepped wedge design, where, upon completion, all participating sites have implemented the intervention, could be a major implementation driver by itself [21]. We therefore do not know how wider scale up of the OASI-CB would fare without OASI1’s substantial infrastructure and support and outside of a research setting.
4. Debate around the OASI-CB: although not a limitation of the OASI1 project itself, the OASI-CB has attracted some debate from professional groups, with criticism pertaining primarily to the quality of the evidence supporting the selected components, and the perception that the bundle may cause women unnecessary distress [22]. These comments stimulated lengthy discussions within the Project Team and with the project’s IAG, which concluded that modification of the bundle’s four core elements is unwarranted. The Project Team has responded directly to some of the concerns raised, citing that the care bundle was developed using the best-available evidence and with input from both clinicians and women [23]. Other misconceptions, such as the care bundle promoting routine episiotomy or limiting women’s birth choices, are clarified by the care bundle FAQs on the project’s webpage [24]. Comments from others have also led to a refinement of the language used to describe the components to emphasise that women’s informed consent must always precede any intervention. The experience of addressing criticisms about the care bundle exemplifies the importance of transparency and clear communication in the development and implementation of any new intervention.

## Improving maternity services: lessons learnt from the OASI1

- Comparable approaches to preventing OASI have been implemented in other countries, including various training programmes and care bundles in Norway [7, 8, 10] and the WHA Perineal Protection Bundle in Australia [25]. The OASI1 Project not only evaluated the OASI-CB’s clinical effectiveness but also identified key barriers and enablers to the implementation of such interventions.
- Selection of OASI-CB elements prioritised easily implementable practices that require no additional supplies or finances to carry them out, making the OASI-CB compatible for implementation not only in the UK but in other health systems globally, with varying resource constraints.
- Strong partnership between national organisations such as the RCOG and RCM (representing obstetric healthcare professionals in UK maternity units) is a key enabler in the implementation of QI interventions.
- The challenge of different QI initiatives competing for attention in busy maternity units is a key barrier to project set-up, especially where other initiatives or adverse outcomes take priority. Maternity units should consider establishing a limited number of priorities for staff to undertake at any one time, so that concerted efforts can be directed towards them to achieve impactful change.
- A conscious focus on mechanisms of implementation alongside assessment of clinical effectiveness accelerates the transition of knowledge to practice [26]. Detailed reporting of studies with such a dual focus provide a helpful blueprint for other maternity units to learn from and build on, as will be the case with the OASI2 study.

## Future directions: OASI2

The amalgamation of identified strengths, limitations, and general lessons that resulted from OASI1 has set the stage for a unique opportunity to build on this knowledge. We know that the OASI-CB is effective in reducing women’s risk of OASI and in reducing OASI rates; we also know that clinicians and women are generally supportive of it. What we do not know is how to achieve successful scale-up of the OASI-CB. “OASI2” is a follow-on study with a primary focus on studying and comparing more scalable mechanisms of OASI-CB implementation, which may serve to guide wider roll out, nationally and beyond. OASI2 is designed as a two-arm cluster randomised control trial (C-RCT) that will compare two implementation approaches: maternity units in one arm will rely on only “passive support” from the Project Team in the form of a toolkit of resources informed by the OASI1 learnings, whereas units in the other arm will receive this passive support in addition to peer-support from clinicians who have experienced care bundle implementation in OASI1. OASI2 will determine whether these two approaches lead to differing levels of care bundle adoption by clinicians and whether they have an impact on the OASI-CB’s effectiveness in reducing OASI rates.

OASI2 represents a shift in thinking, from whether the OASI-CB can reduce OASI rates when implemented with strong support and leadership, to whether it can be implemented with moderate or limited implementation support and still achieve a significant clinical impact. The latter question is more relevant to the typical publicly funded (NHS or otherwise) maternity unit and what it needs to successfully implement quality improvement initiatives. To this effect, OASI2 will also follow up with a number of maternity units from OASI1, to determine the care bundle’s long-term sustainability once centralised expert support is withdrawn. Together, these evaluations will produce evidence for what can be achieved with more modest resources.

## Abbreviations

OASI: Obstetric anal sphincter injury
QI: Quality improvement
RCOG: Royal College of Obstetricians and Gynaecologists
RCM: Royal College of Midwives
OASI-CB: OASI Care Bundle
LSHTM: London School of Hygiene and Tropical Medicine
PPI: Patient and public involvement
MPP: Manual perineal protection
IAG: Independent advisory group
